# Is poverty the mother of crime? Evidence from homicide rates in China

**DOI:** 10.1371/journal.pone.0233034

**Published:** 2020-05-18

**Authors:** Baomin Dong, Peter H. Egger, Yibei Guo

**Affiliations:** 1 Center for Yellow River Civilization and Sustainable Development, and School of Economics, Henan University, Kaifeng, Henan Province, China; 2 Department of Management, Technology and Economics, ETH Zürich, Zürich, Switzerland; 3 Institute of Economics and Management, Henan Agricultural University, Zhengzhou, Henan Province, China; Xiamen University, CHINA

## Abstract

Income inequality is blamed for being the main driver of violent crime by the majority of the literature. However, earlier work on the topic largely neglects the role of poverty and income levels as opposed to income inequality. The current paper uses all court verdicts for homicide cases in China between 2014 and 2016, as well as various inequality measures calculated from 2005 mini census data together with a host of control variables to shed light on the relationship at the detailed Chinese prefecture-level. The results suggest that it is the poverty and low income level, rather than income inequality, that is positively related to homicide rates. We show that the internal rural-urban migration from more violent localities contributes to the destination cities’ homicide rates. The poverty-homicide association implies that instead of “relative deprivation”, “absolute deprivation” is mainly responsible for violent crime. Poverty is the mother of crime. —Marcus Aurelius (121-180AD), Emperor of the Roman Empire.

## Introduction

On June 22, 2017, a mother and her three children died in their home in a tragic fire set deliberately by the family’s migrant worker nanny on the 18th floor of a luxurious high-rise building in Hangzhou, China. The high-profile homicide case in Hangzhou had quickly become a focus of attention for Chinese netizens, with over 200 million views on the Weibo blog within four days. The victim family’s wealth and the nanny’s impoverished and migrant background triggered a nationwide debate on violent crime and tensions over China’s poor-rich divide.

Needless to say, violent crimes such as homicide negatively affect economic development. World Bank [1] reports that decreasing 10% of a country’s homicides would lift up per capita GDP by 0.7% to 2.9% over the subsequent five years even after controlling for a variety of other determinants. The homicide rate is also an important indicator for rule-of-law [2]. Homicide is an important dimension of disamenity and can be severely underestimated if the model formulation is incorrect [3, 4]. High homicide rates also lower citizens’ satisfaction on democracy, which consequently impair further democratic institutions and economic development [5]. Policy-makers around the world enact laws to impact public safety, and a controversial one is ‘Stand Your Ground’ on the use of gun violence [6].

There is a growing literature arguing that income inequality causes crime, beginning with Becker [7]. The mechanisms seem to be straightforward. For instance, the theory of ‘relative deprivation’ introduced by Merton [8] suggests that income inequality strengthens the feelings of dispossession and unfairness. The inequality-crime association that empirically identified has been widely accepted. For instance, Messner and Rosenfeld [9] state that a ‘finding that has emerged with remarkable consistency is that high rates of homicide tend to accompany high levels of inequality’, and solidified in both intra-national and cross-national studies, but it is not without debate.

A fundamental debate centers on whether poverty or inequality drives violent crime. Although many studies show a positive relationship between income inequality and the rate of violent crime, some critical studies using similar data argue that when poverty is added to the regressions, income inequality is not significant anymore and (absolute) poverty explains the crime rate, while (relative) income inequality does not.

Another criticism of the ambiguous effect of inequality on crime in cross country studies is that controlling confounding factors at the country level is inherently difficult. Neumayer [10] argues that by increasing the sample size of countries in Fajnzylber, Lederman, and Loayza [11], the Gini coefficient is no longer statistically significant in predicting violent crime. On top of the lack of observability of confounders, income inequality measures for different countries are based on different income concepts and definitions change which places a measurement bias problem, as demonstrated by Atkinson and Brandolini [12].

In terms of possible reverse causality between income measures (such as various measures of income inequality) and crime rates, the existing literature usually employs an instrumental variable approach but seldom defend the relationship from an intuitive perspective. Nevertheless, it is often argued that the well-offs are able to invest in protection or preventative measures, including installing surveillance system, moving to better communities, and owning a car, etc., thus the reverse causality between property crime and income inequality is also weakened by such argument. In fact, it is documented by Levitt [13] that households with low income ($25,000 and below) suffer a 60% more chance from burglar than high-income ones ($50,000 and above).

In one study dedicated to the possible reverse causality between crime and inequality, Barenboim [14] shows that although theoretical model predicts that the rich would spend more on prevention, the empirical evidence from the US indicates that the poor use more expensive transportations to work in areas with the higher violent crime rate. Barenboim [14] finds a small positive effect of property crime on inequality, but no evidence is shown for such an association between homicide and inequality to exist, to the best of our knowledge. Given that homicide rate is low in absolute magnitude and homicide is largely non-random, it is uneconomic to relocate oneself due to a higher city-level homicide rate.

Another related and commonly believed important factor for violent crimes such as homicide beyond poverty and inequality is immigration. The empirical literature on cross country immigration finds little support of a positive association which runs in contrast to the contrary theory which considers immigration to segregate society and, hence, to stimulate crime. The empirical results seem to support the positive theory, which argues that the fear of deportation, together with the appreciation for a better life often motivate immigrants more than the locals to honor the law. However, the effect of internal migration on local crime rates is understudied by the literature partly due to the lack of data. One exception is Caminha et al. [15], who find cities with a higher floating population have higher rates of property crimes.

China offers a unique testing ground for the mobility of people and crime for its humongous size of internal migration. According to China Labour Bulletin [16], there were an estimated 287 million rural migrant workers in China in 2017, making up more than one third of the entire working population. The perception that migrants contribute to most of the crimes may not be mistaken in China. According to a recent popular media analysis [17], migrants make up over 80% of total violent crimes but the official statistics do not include such information. The Study Group of Xiamen Public Safety Department [18] also shows that in Chinese cities, the crime rates of immigrants are five to six times higher than those of local hukou residents. However there is a lack of scholarly research on the impact of intra-national migration on violent crimes such as homicide.

The current paper studies the relationship between income measures and homicide rate by using unique data on violent crimes in China, together with mini-census data which give information on the prefecture-level income distribution (allowing to define inequality and poverty rates). We use the “reduced form” model to identify the causality between income measures and homicide rates. Moreover, we use internal migration data between prefectures to control for the violence from migrants. Our results suggest that it is poverty and the income level per se, rather than income inequality, that contributes to the homicide rate. While the rural area poverty level works as a push factor for homicide in a prefecture, boosting crime there, higher average income in urban areas appears to work as a pull factor, attracting crime in the prefecture as a whole.

We believe that the evidence from China in this paper, due to the regionally much more detailed (prefecture-level rather than province-level) account of the dependent and independent variables involved, adds to the ongoing debate on the roles of different dimensions of income and migration for violent crime.

The rest of the paper is organized as follows. Section 2 gives a brief review of the literature. Section 3 describes data and empirical strategy. Section 4 discusses the results. Section 5 concludes the paper.

## Literature review

Homicide, as the intentional killing of a person by another, represents the most serious criminal offense in all violent crimes. It is the most widely collected and reported crime in law enforcement and criminal justice statistics. Compared with other types of crime, homicide is least likely to be biased in measurement (e.g., [19–21]). By contrast, e.g., only two-thirds of all burglaries are recorded by the police from victim surveys and police-recorded crime in England and Wales [22].

A burgeoning literature attempts to explain violent crimes such as homicide by income inequality. The vast majority of the studies show a positive relationship, such as the 34 cross-sectional studies surveyed in Hsieh and Pugh [23] and the original papers by Lederman et al. [24], Imrohoroglu et al. [25], Soares [26], and Pickett et al. [27], etc. Notable examples include Blau and Blau [28] who find such a relationship to exist in the US and Kelly [29] who concludes that robbery, assault, and the aggregate level of crime are all influenced by income inequality. Messner et al. [30] find a positive relationship between the Gini coefficient and homicides in the US. Recent evidence for a positive relationship between inequality and the homicide rate is found by Fajnzylber et al. [11, 31] for a cross-section of industrialized and developing countries; Poveda [32] for seven major cities in Colombia; Nadanovsky and Cunha-Cruz [33] for Latin America; and Demombynes and Ozler [34] for South Africa.

However, Mathur [35] finds that the Gini coefficient had an ambiguous effect on crime, and Stack [36] finds no relationship using data from Interpol for a cross-section of countries. Neumayer [10] directly questions the results in Fajnzylber et al. [11] since by increasing the sample size of countries, the Gini is no longer significant in explaining violent crime. Guillaumont and Puech [37] also do not find a significant impact of income inequality on crime rates. Some other studies, such as Brush [38] and Chintrakarn and Herzer [39], even find a negative relationship between income inequality and crime in the US. A common interpretation for this seemingly counterintuitive result is that the larger inequality triggers a larger demand for security devices or services, which leads to a reduction of crime rate. Costantini et al. [40] report a mixed message in which both inequality and unemployment rate positively impact violent crime where unemployment is often regarded as a proxy of absolute poverty.

Indeed, Pridemore [41] argues that the positive inequality-homicide association may be a spurious result of model misspecification. In particular, he argues that most cross-national studies of homicide fail to control for poverty, which is the most consistent predictor of area homicide rates in the US empirical literature. In fact, empirical studies implied a poverty-homicide association, e.g., D’Ambrosio and Rodrigues [42] find a strong spatial correlation between homicide and favelas concentration in Latin America. It is argued that the poverty-homicide association works through disintegrating individuals from society, making them prone to commit to violent crimes [43].

For the case of China, a number of studies have been carried out to examine the relationship (e.g., [44–53], etc.). Most of them find an inequality-crime association. However, virtually all of the existing papers suffer from problems of small sample size, the use of regionally very aggregated data, the use of aggregated rather than specific types of crime, and relatively imprecise measurement of inequality.

For instance, most of the studies use province-level data, so that the number of observations is small and there is a risk of spatial aggregation bias (income may be relatively equal within but large between meso-regions within provinces, and the relationship to crime within a province may be spurious). Moreover, almost all of the studies took crime data from China Procuratorial Yearbooks in which only aggregate numbers of arrests in each province are reported. This brings multiple difficulties that are hard to overcome. E.g., with aggregate numbers of all arrests, the impact of inequality on specific types of crime may not be studied. The aggregation across different crime types may be of particular concern here, as the relationship between inequality and violent crime depends on the type of crime. For instance, some studies find that income inequality affects homicide rates but not on other types of violent crime such as rape or assault [54–56]. The use of data from China Procuratorial Yearbooks may be a problem since under-reporting [57] or under-registration [58] of murder and other violent crimes by the police is a widespread issue.

Regarding the measurement of income inequality, most papers use the urban-rural divide to represent provincial income inequality but its impact on violent crime is hard to justify. E.g., Shi and Wu’s [51] ‘regional disparity’ is merely the difference between the national average income and provincial income. The most ‘accurate’ estimate of inequality in this line of research, is the provincial Gini coefficient calculated from five quantiles of income grouping recorded in the provincial statistical yearbooks. Similar problems exist for control variables. For instance, the China Procuratorial Yearbooks only give total expenditures of public security agency (police), the procuratorial offices, and the court and judicial agency together, whereas only expenditures on police should directly deter crime. Hence, it is unclear whether counterintuitive results or a lack of significance of the findings should be attributed to the absence of the actual effect, the small sample size, or the aforementioned measurement problems.

Another strand of the literature looks into the relationship between immigration and crime. Despite the widespread perception that immigration is responsible for violent crimes such as homicide, scholarly research finds little empirical evidence for that (see, e.g., in Papadopoulos [59]). Indeed, numerous studies find that higher concentrations of immigrants are associated with lower crime rates (e.g., Pendergast et al. [60]). Some studies even establish causality to show that immigration led to lower crime rates. Historically, Sequeira, Nunn and Qian [61] show that the Age of Mass Migration (1850-1920) had no long-run effects on crime rates in the US. It is also argued that the positive relationship between immigrants and crime in some countries may be due to racial or ethnic discrimination by the police and the judicial system.

However, little is known for the effect of a country’s internal migration on local crime rates. As an exception, Cheng, Liu, and Wang [62] combine the arrest and prosecution data from 306 prefectures in China with interviews with nine policemen and public procurators from five provinces to show that the ratio of migrants (in the total population) contributes more to the prosecution rate whereas the ratio of home rental over homeownership impacts more on the arrest rate. Thus the implication is that internal migration introduces crime and part of it is carried through the rental-housing channel. In the Chinese language literature, Chen, Li, and Chen [63] argue that the rise of large-scale internal migration in China is the main reason for the increase in crime rates. However, some other studies, e.g., Tong [64] finds that migrants are more likely to be victims than criminals due to their low social status and exposure to a complicated environment, etc.

## Data and empirical model

The aforementioned data aggregation problem and under-reporting in official statistics make officially published data not well suited for research. However, the movement toward transparency of conviction inflicted by China’s top leader in 2013 resulted in the unconditional disclosure of all court verdicts since January 1, 2014. By decree of the Supreme Court of China, all court verdicts must be made available to public online unconditionally within seven days of judgment, with exceptions of cases of juvenile accused or of national security concerns, beginning by January 1, 2014. Verdicts are collected from PKULaw.cn (http://www.pkulaw.cn/) which is the largest and most inclusive database for legal documents in China. We hand-collected first-instance judgments of all homicide cases from January 1, 2014, to December 31, 2016, from all levels of courts in China, and aggregated them by type to the prefecture level, to form the dependent variable. Thus our data overcomes the incompleteness problem of official statistics, allows a precise measurement of violent crime, and can be measured at the prefecture level rather than province level.

### Data

In this study, we use data from PKULaw.cn on all homicides at the level of prefectures that were treated in Chinese courts in 2014 or, alternatively, in 2014-2016. Since the convictions do not reveal any personal information of the victims or the convicted, including second- and/or third-instance information would only bias the data, then we only use the first-stance court convictions and there were in total 8,354 first-stance court convictions in the homicide category between January 1, 2014, and December 31, 2016, published by PKULaw.cn. We normalize the respective numbers by the population size in each prefecture, to obtain the prefecture-level homicide rate. We dub the respective variable *Homicide*. In general, as the explanatory variables are not available at an annual level for this time span, we will use index *i* to denote prefectures and use data for 2014 or averages for the years 2014-2016.

We use the following set of explanatory variables to explain *Homicide*_*i*_ across prefectures.

#### Income distribution and poverty

First of all, we use *Poverty*_*i*_, to measure the fraction of the population in the prefecture *i*, which has an income below the fifth percentile of the overall income distribution of China. Furthermore, we also consider the effect of *PovertyUrb*_*i*_ and *PovertyRur*_*i*_ in the urban and rural areas of the prefecture *i* on *Homicide*_*i*_, respectively. Data to compute income percentiles for all of China and for each prefecture underlying the respective poverty variables and variables introduced below are from China’s 2005 mini census of 1% of the population, which is the only dataset with a large-enough sample size to construct China’s prefecture-level income inequality measures. In the 2005 mini census, we use the place of residence (not according to the Hukou type) to define the rural and urban areas. A positive parameter on one of these poverty variables would indicate that an increase in the number of people below the poverty line (in urban or rural areas) would increase the homicide rate in a prefecture.

Moreover, we employ various income-level and income-inequality measures. First of all, we include the log of average income *LnAvgInc*_*i*_, as well as log of average income in urban areas, *LnAvgUrbInc*_*i*_, and rural areas, *LnAvgRurInc*_*i*_, as three separate measures of income in a prefecture. A positive parameter on the average income variables would indicate that an increase in average income (in urban or rural areas) would increase the homicide rate in a prefecture.

Second, we employ three measures of income inequality, all of them also defined for urban and rural areas separately, pertaining to different brackets of the income distribution: (1) the log difference between the 95th and the 5th percentiles, *DLnInc*9505_*i*_, *DLnUrbInc*9505_*i*_, and *DLnRurInc*9505_*i*_; (2)the log difference between the 90th and the 10th percentiles, *DLnInc*9010_*i*_, *DLnUrbInc*9010_*i*_, and *DLnRurInc*9010_*i*_; and (3) the log difference between the 75th and the 25th percentiles, *DLnInc*7525_*i*_, *DLnUrbInc*7525_*i*_, and *DLnRurInc*7525_*i*_. While the first measure indicates the income gap in the very tail of the distribution, the last measure captures the interquartile range. The three measures are considered together in order to permit a potentially nonparametric impact of income inequality on *Homicide*_*i*_. A positive parameter on one of these income dispersion variables would indicate that an increase in income dispersion of the respective kind (in urban or rural areas) would increase the homicide rate in a prefecture, even after conditioning on average income and poverty levels per urban or rural area in the prefecture.

We generate the Gini coefficient *Gini*_*i*_ for the prefecture *i* as another measure of inequality between urban and rural area in one additional robustness check. Data for calculating the Gini coefficient is also based on 2005 mini census. A positive coefficient on *Gini*_*i*_ would indicate that an increase in income dispersion between urban and rural areas would increase the homicide rate in a prefecture, and such a result is consistent with results using other income inequality measures.

#### Demography and employment

We employ the following variables and sources to capture demographic factors. *LnPopUrb*_*i*_ and *LnPopRur*_*i*_ are the log resident population numbers in urban and rural areas of a prefecture. The data source for these variables is CEIC (www.ceicdata.com). Moreover, we use the overall population of a prefecture and divide it by the respective area size (in squared kilometers) to obtain population density data, *PopDens*_*i*_. At least for the United States there is evidence that economic crimes are relatively more frequent in more densely populated areas.

Moreover, we employ the ratio of college students in the total population of a prefecture, *CollRate*_*i*_, the unemployment rate, *UnempRate*_*i*_, and the share of manufacturing employment in the total population of a prefecture, *IndEmpRate*_*i*_, all using data from CEIC. Education is also considered in the empirical models since it increases employability, thus decreasing the probability of committing to a crime. Higher levels of unemployment provide direct incentives for economically driven crimes, as justified by Becker [7], Ehrlich [65], and Chiu and Madden [66]. However, we do not have a clear-cut reference point regarding the frequency of homicides in microregions with a higher versus a lower college or unemployment rate, at least not for China.

#### Policing, jurisdiction, and violent environment

Public security expenditures are hand-collected from all prefecture city governments’ websites on the final settlement of budgetary expenditures for the year 2014. We use the log of prefecture-level public security expenditure and dub it *LnPolicing*_*i*_. Earlier work on crime identified a positive impact of policing on crime numbers, as more policing makes detection and arrests of criminals easier [7]. Although more policing activities may also deter crimes, the existing literature found no such effect on violent crimes [29]. Moreover, we employ the ratio of arrests and convictions, *ArrConv*_*i*_. Notice that convictions pertain to a potentially different base of arrested individuals than arrests do. Hence, the respective variable is not necessarily larger than unity. However, in the long run, we expect a larger value of *ArrConv*_*i*_ to reflect a less severe threat of the court system for criminals, all else equal. Hence, we expect a positive influence of this variable on *Homicide*_*i*_.

Based on *N* prefectures, and *P* provinces, we make twofold use of the *N* × *P* matrix of immigration into (cities in) prefectures and the *N* × 1 vector of intentional injury cases across prefectures from PKULaw.cn. First, using the population distribution across cities, prefectures, and provinces from CEIC data and assuming proportionality about the immigration across prefectures from provinces into cities from the China Migrants Dynamic Survey, we impute an *N* × *N* prefecture-to-prefecture immigration matrix where the lines are destinations and the columns are origins. Let us call a typical entry of that matrix *m*_*ij*_, where *i* is a destination and *j* an origin. Following customary practice with neighbor weighting in the spatial econometrics literature (see, e.g., Kelejian and Prucha [67]), we normalize *m*_*ii*_ = 0 for all prefectures *i*. Moreover, let us refer to *ι*_*j*_ as a typical entry of the vector of prefecture-level intentional injury cases. Then, we generate the variable *NeighborViolence*_*i*_ based on $\sum_{j=1}^{N}\frac{m_{ij}\iota_{j}}{\sum_{j=1}^{N}m_{ij}}$. Hence, *NeighborViolence*_*i*_ is the immigration-weighted number of intentional injury cases in other prefectures than a given one. Similarly, we define *ViolentImmig*_*i*_ as $\sum_{j=1}^{N}\frac{\iota_{j}m_{ij}}{\sum_{j=1}^{N}\iota_{j}-\iota_{i}}$, which is the intentional-injury-weighted number of immigrants from other prefectures. For controlling the net inflow poor migrants’ effect on homicide, we generate another variable *ImmigPoor*_*i*_ based on the immigration matrix and the number of residents living in the minimum subsistence allowances in 2014 from statistical yearbook of each prefecture. We define *ImmigPoor*_*i*_ as $\sum_{j=1}^{N}m_{ij}\omega_{j}-\sum_{i=1}^{N}m_{ji}\omega_{i}$ to resprensent the net inflow of poor migrants to prefecture *j*, where *ω*_*j*_ and *ω*_*i*_ are the proportion of the residents living in the minimum subsistence allowances in prefecture *j* and prefecture *i*.

A positive parameter on *NeighborViolence*_*i*_ would indicate that homicides are higher in prefectures which are located in the vicinity of prefectures with high rates of intentional injuries. Hence, this variable reflects one source of potential cross-border spillover effects of violent crime. A positive parameter on *ViolentImmig*_*i*_ would indicate that homicides are higher in prefectures which are more important destinations of migrants from prefectures with high rates of intentional injuries. Hence, this variable reflects another source of the potential cross-border spillover effect of violent crime. A positive parameter on *ImmigPoor*_*i*_ would indicate the homicides are higher in prefectures which had more net inflow of migrants. By adding this variable, we are able to identify whether poverty imported via inflows of migrants contributed to local violent crime.

#### Region fixed effects

Apart from these continuous variables, we also include binary indicator variables for seven geographical divisions often used to aggregate Chinese provinces. These regions are: Eastern China (Anhui, Fujian, Jiangsu, Jiangxi, Shandong, Shanghai, Zhejiang); Southern China (Guangdong, Guangxi, Hainan); Northern China (Beijing, Hebei, Inner Mongolia, Shanxi, Tianjin); Central China (Henan, Hubei, Hunan); Northeastern China (Heilongjiang, Jilin, Liaoning); Southwestern China (Chongqing, Guizhou, Sichuan, Tibet, Yunnan); Northwestern China (Gansu, Ningxia, Shaanxi, Xinjiang).

#### Descriptive statistics

We provide a list of the variables just introduced together with a short definition and descriptive statistics in Table 1. For each variable used, we provide the number of prefectures this variable would be available for (*Obs*.), the average value the variable takes on (*Mean*), as well as the corresponding standard deviation (Std.dev.), the minimum (*Min*.), and the maximum (*Max*.).

**Table 1:** Summary statistics.

Variable	Definition	Obs	Mean	Std.Dev.	Min.	Max.
*Homicide*	prefecture level homicide rate	281	0.016	0.012	0.002	0.083
*Poverty*	share of the poor people	283	0.083	0.035	0.050	0.217
*PovertyUrb*	share of the poor people (urban)	283	0.084	0.034	0.050	0.256
*PovertyRur*	share of the poor people (rural)	281	0.087	0.040	0.050	0.246
*LnAvgInc*	log of average income	284	6.352	0.354	5.664	7.583
*LnAvgUrbInc*	log of average income (urban)	284	6.729	0.269	5.852	7.583
*LnAvgRurInc*	log of average income (rural)	284	6.183	0.308	5.580	7.149
*DLnInc*9505	*ln*(*income*_95%_) − *ln*(*income*_5%_)	284	2.075	0.223	1.482	2.659
*DLnUrbInc*9505	*ln*(*income*_95%_) − *ln*(*income*_5%_) (urban)	284	1.984	0.275	1.163	2.813
*DLnRurInc*9505	*ln*(*income*_95%_) − *ln*(*income*_5%_) (rural)	284	1.943	0.235	1.079	2.708
*DLnInc*9010	*ln*(*income*_90%_) − *ln*(*income*_10%_)	284	1.679	0.221	0.981	2.303
*DLnUrbInc*9010	*ln*(*income*_90%_) − *ln*(*income*_10%_) (urban)	284	1.579	0.248	0.956	2.303
*DLnRurInc*9010	*ln*(*income*_90%_) − *ln*(*income*_10%_) (rural)	284	1.565	0.223	0.742	2.303
*DLnInc*7525	*ln*(*income*_75%_) − *ln*(*income*_25%_)	284	0.919	0.197	0.379	1.386
*DLnUrbInc*7525	*ln*(*income*_75%_) − *ln*(*income*_25%_) (urban)	284	0.861	0.184	0.357	1.504
*DLnRurInc*7525	*ln*(*income*_75%_) − *ln*(*income*_25%_) (rural)	284	0.828	0.189	0.322	1.386
*LnPolicing*	log of prefecture-level public security expenditure	284	7.292	0.807	4.631	9.940
*ArrConv*	ratio of arrests and convictions	284	0.671	0.343	0.243	5.007
*NeighborViolence*	Neighbor’s weighted number of intentional injury cases	276	5.957	0.495	5.085	7.467
*ViolentImmig*	intentional-injury-weighted number of immigrants	276	0.073	1.277	-2.429	3.857
*LnImmiPoor*	log of net inflow poor migrants	276	2.900	1.961	-2.615	4.229
*LnPopUrb*	log population (urban)	284	0.051	0.775	-1.877	2.967
*LnPopRur*	log population (rural)	284	1.250	0.693	-1.528	3.460
*UnempRate*	unemployment rate	278	0.006	0.006	0.000	0.051
*CollRate*	fraction of college students	280	0.021	0.038	0.000	0.363
*IndEmpRate*	share of manufacturing employment	281	0.044	0.070	0.001	0.786
*PopDens*	population density	284	7.053	0.813	4.789	10.138
*Gini*	Gini coefficient	284	0.389	0.040	0.286	0.507
*GiniUrb*	Gini coefficient (urban)	284	0.344	0.050	0.221	0.509
*GiniRur*	Gini coefficient (rural)	282	0.371	0.048	0.000	0.469
*Region*	seven regions for Chinese provinces	284	3.448	2.073	1	7

We will suppress a lengthy discussion of the descriptive statistics for the sake of brevity but will highlight a few facts. The degrees of poverty vary widely across different prefectures in China, as well as average income and income inequality also show regional differences. The apparent contrast of poverty and income inequality also can be seen when we consider urban and rural areas separately. First of all, average urban income is higher than average rural income, as can be seen from the average values of the variables *LnAvgUrbInc* and *LnAvgRurInc*. Moreover, the average inequality is higher in urban than in rural areas for any inter-quantile range considered. To see this, compare the average values of *DLnUrbInc9505*, *DLnUrbInc9010*, and *DLnUrbInc7525* with those of *DLnRurInc9505*, *DLnRurInc9010*, and *DLnRurInc7525*, respectively. Observe also that the average rural poverty is higher than its urban counterpart (see the statistics for *PovertyUrb* vs. *PovertyRur*).

Urban areas tend to be smaller than rural ones according to the averages of *LnPopUrb* and *LnPopRur*, but the largest urban area is larger than the largest rural one across the prefectures (see the maxima of the same variables).

Finally, consistent with our earlier discussion, the ratio of arrests to convictions of crimes is not bounded by unity from below. To see this, consider the maximum value of *ArrConv* in Table 1.

### Empirical model

Generally, the dependent variable and covariates may be endogenous due to simultaneity, and it shall be treated with care. In the case of the lack of convincing instrumental variables, a reduced form regression using lagged variables is suggested by the literature. Following Angrist and Pischke [68], we use a reduced-form model to identify the causality. Suppose we have such structure model with the true regression coefficient we are interested in:

$$\begin{array}{c} E\left({Homicide}_{i,t}|X_{i,t}\right)=G\left(X_{i,t}\gamma \right)W_{i,t},\;i=1,...,N;t=2014 \end{array}$$ (1)

where indices *i* for prefectures, *t* for year, vector *X*_*i*,*t*_ is an endogenous variable, and *W*_*i*,*t*_ is the disturbance. Consider using the poverty and inequality measure in 2005 as instrumental variables, which is the lag of our endogenous income measures. Because the lag of income measures affects the current term directly, and cannot play any role in the current homicide rate, the true structure model in the first stage is then:

$$\begin{array}{c} E\left(X_{i,t}|X_{i,t-1}\right)=G\left(X_{i,t-1}\alpha \right)U_{i,t-1},\;i=1,...,N;t=2005,2014 \end{array}$$ (2)

Thus the reduced form is the regression of the dependent variable on the instrument *X*_*i*,*t*−1_ given by:

$$\begin{array}{c} E\left({Homicide}_{i,t}|X_{i,t-1}\right)=G\left(X_{i,t-1}\beta \right)U_{i,t},\;i=1,...,N;t=2005,2014 \end{array}$$ (3)

where *U*_*i*,*t*_ is the disturbance. Assuming *E*(*X*_*i*,*t*−1_ *W*_*i*,*t*_) = 0, that is, the lag of the poverty and inequality affects homicide rates only through affecting themselves in 2014, and *E*(*X*_*i*,*t*−1_ *X*_*i*,*t*_)≠0. It is credible to assert that the income measures in the past can be proper IVs to affect homicide rates in the current.

To reduce the notational burden, we eliminate the subscript of year *t* and include all the aforementioned prefecture-level variables including our IV into the vector *Y*_*i*_. Using *a* for seven geographical divisions (in which prefectures are nested) and denote region fixed effects by *μ*_*a*_, we can write the empirical model we estimate as:

$$\begin{array}{c} E\left({Homicide}_{i}|Y_{i},\mu_{a},U_{i}\right)=G\left(Y_{i}\beta +\mu_{a}\right)U_{i},\;i=1,...,N \end{array}$$ (4)

where *G*(·) indicates that we estimate a generalized-linear exponential-family model to accommodate the non-negativity of the dependent variable *Homicide*_*i*_, and *U*_*i*_ is the respective disturbance term which obeys *E*(*U*_*i*_) = 1 and *U*_*i*_ ≥ 0 and which we treat as potentially heteroskedastic. As *Homicide*_*i*_ ∈ [0; 1) is defined as a rate that is bounded from below, and from above, we follow Papke and Wooldridge [69] and use a fractional-response framework. Typically, *G*(·) rests on a logistic function *G*(*z*) = exp(*z*)/(1 + exp(*z*)), which maps *z* to the(0, 1) interval. Then we maximize the binomial log likelihood with individual contribution given by:

$$\begin{array}{c} \ell_{i}\left(\beta \right)={Homicide}_{i}\log\left[G\left(Y_{i}\beta +\mu_{a}\right)\right]+\left(1-{Homicide}_{i}\right)\log\left[1-G\left(Y_{i}\beta +\mu_{a}\right)\right]. \end{array}$$ (5)

One point noteworthy in the reduced form regressions is that the effect might be biased because the income level (or income inequality) in 2005 may have had an effect on 2014 income levels. Then the coefficient (***β***) here contains the whole effect which includes direct effect (***γ***) and indirect effect (***α***) of income inequality in 2014 on crime rates in 2014. From the above model, we may estimate coefficients with upward bias, however, the reduced form does not change the direction of the causality. At the same time, using the income measures in 2005 as an IV alleviates the problem of endogeneity due to simultaneity.

## Results

We put regression results in five tables. Tables 2 and 3 present the results at the whole prefecture level. Table 4 presents the results in which the relevant income measures are calculated at prefectural urban and rural levels. Table 5 reports robustness checks where inequality measures are substituted by Gini coefficients.

**Table 2:** The effect of income inequality on homicide.

	*Dependent variable: Homicide*						
	(1)	(2)	(3)	(4)	(5)	(6)	(7)
*DLnInc*9505	-0.283	-0.064					-0.521*
	(2.589)	(2.792)					(0.279)
*DLnInc*9010			0.004	0.246			0.718**
			(0.260)	(0.179)			(0.335)
*DLnInc*7525					0.057	0.027	-0.224
					(0.294)	(0.205)	(0.295)
*Lnpolicing*	-0.169	0.022	-0.168**	0.029	-0.167**	0.024	0.023
	(0.789)	(1.030)	(0.070)	(0.062)	(0.070)	(0.062)	(0.062)
*ArrConv*	-0.461	-0.163	-0.441***	-0.143	-0.439***	-0.155*	-0.180*
	(2.384)	(2.132)	(0.171)	(0.096)	(0.169)	(0.094)	(0.099)
*NeighborViolence*	-0.096	-0.033	-0.072	-0.016	-0.072	-0.030	-0.022
	(1.411)	(1.477)	(0.097)	(0.088)	(0.098)	(0.089)	(0.092)
*ViolentImmig*	0.200	0.197	0.185***	0.190***	0.185***	0.195***	0.205***
	(0.614)	(0.706)	(0.052)	(0.042)	(0.051)	(0.042)	(0.043)
*LnImmigPoor*	0.001	0.022	0.006	0.030	0.008	0.024	0.029
	(0.311)	(0.348)	(0.032)	(0.020)	(0.033)	(0.020)	(0.020)
*LnPopUrb*		-0.015		-0.010		-0.017	0.012
		(1.187)		(0.077)		(0.077)	(0.073)
*LnPopRur*		-0.540		-0.543***		-0.538***	-0.567***
		(1.324)		(0.087)		(0.084)	(0.080)
*UnempRate*		13.873		15.928		14.413	14.490
		(12.830)		(12.194)		(12.292)	(10.251)
*CollRate*		-1.409		-1.839		-1.527	-1.502
		(20.323)		(1.373)		(1.354)	(1.391)
*IndEmpRate*		0.967		1.031**		0.981**	1.030**
		(8.323)		(0.435)		(0.402)	(0.413)
*PopDens*		0.152		0.155***		0.150**	0.178***
		(0.814)		(0.059)		(0.060)	(0.056)
Region FE	Yes	Yes	Yes	Yes	Yes	Yes	Yes
LR *p-value*	0.00	0.00	0.00	0.00	0.00	0.00	0.00
*χ*^2^(*p-value*)	0.05(1.0)	0.01(1.0)	0.02(1.0)	0.02(1.0)	0.02(1.0)	0.01(1.0)	0.02(1.0)
*N*	272	263	272	263	272	263	263

We report the *p-value* of LR test and *χ*^2^ value (*p-value* in parentheses) of H-L test in the last two rows. Robustness standard errors in parentheses;

* *p* < 0.1,

** *p* < 0.05,

*** *p* < 0.01.

**Table 3:** The effect of poverty level and income inequality on homicide.

	*Dependent variable: Homicide*						
	(1)	(2)	(3)	(4)	(5)	(6)	(7)
*Poverty*	2.736**		1.950*	1.026	0.794	1.112	-0.707
	(1.185)		(1.142)	(1.180)	(1.177)	(1.178)	(1.421)
*LnAvgInc*		0.829***	0.793***	0.151	0.101	0.144	0.180
		(0.148)	(0.143)	(0.197)	(0.198)	(0.205)	(0.200)
*DLnInc*9505				-0.056			-0.653*
				(0.174)			(0.353)
*DLnInc*9010					0.201		0.818**
					(0.178)		(0.390)
*DLnInc*7525						-0.027	-0.246
						(0.212)	(0.301)
*Lnpolicing*	-0.156**	-0.186***	-0.176***	0.012	0.022	0.014	0.013
	(0.070)	(0.059)	(0.059)	(0.061)	(0.062)	(0.061)	(0.061)
*ArrConv*	-0.477***	-0.279**	-0.305**	-0.167*	-0.151	-0.165*	-0.161
	(0.180)	(0.129)	(0.134)	(0.100)	(0.099)	(0.099)	(0.099)
*NeighborViolence*	-0.046	-0.003	0.011	-0.006	0.001	-0.001	-0.018
	(0.099)	(0.098)	(0.098)	(0.093)	(0.091)	(0.094)	(0.097)
*ViolentImmig*	0.200***	0.052	0.067	0.183***	0.181***	0.182***	0.182***
	(0.053)	(0.051)	(0.052)	(0.049)	(0.050)	(0.049)	(0.049)
*LnImmigPoor*	0.011	-0.009	-0.006	0.019	0.028	0.020	0.024
	(0.031)	(0.027)	(0.026)	(0.021)	(0.021)	(0.022)	(0.022)
*LnPopUrb*				-0.037	-0.026	-0.036	-0.015
				(0.090)	(0.089)	(0.091)	(0.085)
*LnPopRur*				-0.477***	-0.499***	-0.480***	-0.516***
				(0.114)	(0.115)	(0.116)	(0.109)
*UnempRate*				14.726	16.180	15.024	14.773
				(12.089)	(12.127)	(12.052)	(10.259)
*CollRate*				-1.474	-1.817	-1.550	-1.430
				(1.367)	(1.360)	(1.332)	(1.356)
*IndEmpRate*				0.819*	0.920**	0.826*	0.825*
				(0.428)	(0.469)	(0.436)	(0.430)
*PopDens*				0.151**	0.154***	0.151**	0.171***
				(0.060)	(0.059)	(0.060)	(0.056)
Region FE	Yes	Yes	Yes	Yes	Yes	Yes	Yes
LR *p-value*	0.00	0.00	0.00	0.00	0.00	0.00	0.00
*χ*^2^(*p-value*)	0.04(1.0)	0.02(1.0)	0.01(1.0)	0.01(1.0)	0.03(1.0)	0.01(1.0)	0.02(1.0)
*N*	272	272	272	263	263	263	263

We report the *p-value* of LR test and *χ*^2^ value (*p-value* in parentheses) of H-L test in the last two rows. Robustness standard errors in parentheses;

* *p* < 0.1,

** *p* < 0.05,

*** *p* < 0.01.

**Table 4:** The effect of poverty level and income inequality in rural and urban areas.

	*Dependent variable: Homicide*						
	(1)	(2)	(3)	(4)	(5)	(6)	(7)
*PovertyUrb*	-0.986		-0.047	-0.025	0.579	0.279	-0.734
	(1.234)		(1.185)	(1.196)	(1.109)	(1.028)	(1.339)
*PovertyRur*	2.905***		2.806***	1.958**	2.271***	1.958**	2.637***
	(0.887)		(0.904)	(0.885)	(0.837)	(0.817)	(0.885)
*LnAvgUrbInc*		0.745***	0.683**	0.506**	0.535**	0.477**	0.484**
		(0.259)	(0.271)	(0.209)	(0.215)	(0.210)	(0.216)
*LnAvgRurInc*		0.440**	0.383*	-0.159	-0.109	-0.017	-0.105
		(0.213)	(0.221)	(0.205)	(0.183)	(0.181)	(0.198)
*DLnUrbInc*9505				-0.201			-0.288
				(0.145)			(0.232)
*DLnRurInc*9505				-0.096			0.425
				(0.213)			(0.272)
*DLnUrbInc*9010					-0.109		0.221
					(0.156)		(0.252)
*DLnRurInc*9010					-0.250		-0.159
					(0.190)		(0.259)
*DLnUrbInc*7525						-0.160	-0.120
						(0.202)	(0.253)
*DLnRurInc*7525						-0.644***	-0.789***
						(0.204)	(0.266)
*Lnpolicing*	-0.167**	-0.159***	-0.143**	0.048	0.042	0.073	0.085
	(0.072)	(0.059)	(0.061)	(0.064)	(0.062)	(0.060)	(0.061)
*ArrConv*	-0.387**	-0.307**	-0.291**	-0.167*	-0.166*	-0.191**	-0.210**
	(0.162)	(0.131)	(0.133)	(0.094)	(0.097)	(0.097)	(0.094)
*NeighborViolence*	-0.058	-0.014	-0.003	-0.028	-0.014	0.009	0.015
	(0.098)	(0.099)	(0.100)	(0.090)	(0.092)	(0.092)	(0.092)
*ViolentImmig*	0.165***	0.039	0.042	0.130***	0.130***	0.133***	0.130***
	(0.052)	(0.050)	(0.049)	(0.048)	(0.047)	(0.046)	(0.046)
*LnImmigPoor*	-0.001	-0.011	-0.011	0.013	0.013	0.017	0.022
	(0.030)	(0.027)	(0.027)	(0.022)	(0.022)	(0.022)	(0.023)
*LnPopUrb*				-0.024	-0.031	-0.024	-0.014
				(0.081)	(0.080)	(0.077)	(0.078)
*LnPopRur*				-0.437***	-0.425***	-0.465***	-0.469***
				(0.112)	(0.112)	(0.107)	(0.110)
*UnempRate*				16.131	16.687	16.469	19.355
				(12.621)	(12.770)	(12.324)	(12.270)
*CollRate*				-2.397*	-2.261*	-2.191	-2.581*
				(1.345)	(1.348)	(1.355)	(1.333)
*IndEmpRate*				1.091	0.935	0.310	0.342
				(0.706)	(0.713)	(0.738)	(0.727)
*PopDens*				0.125**	0.129**	0.137**	0.135**
				(0.061)	(0.061)	(0.057)	(0.058)
Region FE	Yes	Yes	Yes	Yes	Yes	Yes	Yes
LR *p-value*	0.00	0.00	0.00	0.00	0.00	0.00	0.00
*χ*^2^(*p-value*)	0.09(1.0)	0.03(1.0)	0.03(1.0)	0.02(1.0)	0.02(1.0)	0.02(1.0)	0.01(1.0)
*N*	270	273	270	261	261	261	261

We report the *p-value* of LR test and *χ*^2^ value (*p-value* in parentheses) of H-L test in the last two rows. Robustness standard errors in parentheses;

* *p* < 0.1,

** *p* < 0.05,

*** *p* < 0.01.

**Table 5:** Robustness check: The effect of GINI coefficient on homicide.

	*Dependent variable: Homicide*				
	(1)	(2)	(3)	(4)	(5)
*Gini*	-2.604**	-0.263	-0.342		
	(1.262)	(0.930)	(0.951)		
*GiniUrb*				-1.399	-0.731
				(0.902)	(0.816)
*GiniRur*				-2.512**	-1.566
				(1.128)	(1.147)
*Poverty*			1.131		
			(1.160)		
*PovertyUrb*				0.398	0.572
				(1.155)	(1.090)
*PovertyRur*				2.682***	2.138***
				(0.859)	(0.827)
*LnAvgInc*			0.136		
			(0.202)		
*LnAvgUrbInc*				0.699***	0.504**
				(0.254)	(0.215)
*LnAvgRurInc*				0.321	-0.137
				(0.196)	(0.176)
*Lnpolicing*	-0.169**	0.022	0.013	-0.137**	0.045
	(0.070)	(0.060)	(0.061)	(0.058)	(0.065)
*ArrConv*	-0.435***	-0.158*	-0.167*	-0.287**	-0.160*
	(0.160)	(0.095)	(0.099)	(0.115)	(0.096)
*NeighborViolence*	-0.092	-0.031	-0.004	-0.056	-0.044
	(0.098)	(0.089)	(0.092)	(0.095)	(0.091)
*ViolentImmig*	0.184***	0.194***	0.182***	0.048	0.131***
	(0.049)	(0.041)	(0.049)	(0.049)	(0.046)
*LnImmigPoor*	-0.006	0.022	0.019	-0.022	0.009
	(0.031)	(0.021)	(0.021)	(0.027)	(0.022)
*LnPopUrb*		-0.019	-0.040		-0.036
		(0.079)	(0.091)		(0.081)
*LnPopRur*		-0.535***	-0.475***		-0.420***
		(0.087)	(0.115)		(0.112)
*UnempRate*		13.836	14.461		14.892
		(12.351)	(12.238)		(12.888)
*CollRate*		-1.452	-1.490		-2.266
		(1.376)	(1.359)		(1.393)
*IndEmpRate*		0.980**	0.849*		0.963
		(0.391)	(0.443)		(0.751)
*PopDens*		0.151**	0.151**		0.129**
		(0.060)	(0.060)		(0.059)
Region FE	Yes	Yes	Yes	Yes	Yes
LR *p-value*	0.00	0.00	0.00	0.00	0.00
*χ*^2^(*p-value*)	0.08(1.0)	0.01(1.0)	0.02(1.0)	0.02(1.0)	0.02(1.0)
*N*	273	264	263	269	260

We report the *p-value* of LR test and *χ*^2^ value (*p-value* in parentheses) of H-L test in the last two rows. Robustness standard errors in parentheses;

* *p* < 0.1,

** *p* < 0.05,

*** *p* < 0.01.

In all regressions we control the region fixed effects. We choose the binomial model structure and the logit link function by using the link test which based on Pregibon’s work [70] to test the rationality of the family model structure for the fractional-response framework. We also perform the Hosmer-Lemmeshow (H-L) test to evaluate the goodness of fit of our model. When the p-value of the test is greater than 0.05, then we cannot reject the null hypothesis that the observed and expected proportions are the same across all quantiles, thus the model fits well. Based on the Liklihood Ratio (LR) test and H-L test, all the estimations we run have a high goodness of fit.

Table 2 has seven numbered columns, which differ in terms of the included income measures as well as other demographic factors. Columns (1)-(6) suggest that a higher income difference between the 95th and the 5th percentiles induces lower homicide rates, but higher income differences between the 90th and the 10th percentiles (*DLnInc9010*), and the 75th and the 25th percentiles (*DLnInc7525*) induce higher homicide rates. Only the latter two income inequality measures provide consistent results with the existing literature even though all these three measures are not statistically significant. When three measures are considered together to show the nonparametric impact of income dispersion on homicides in column (7), only the coefficient of *DLnInc9010* is statistically significant and positive. This indicates that an increase in income dispersion would increase the homicide rate at 90th and 10th percentile income inequality. However as we shall demonstrate later, this inequality-income association may be spurious once absolute poverty is introduced in the regressions.

As for the control variables, we find that a higher arrest-to-conviction rate (*ArrConv*) reduces prefecture-level homicide rates, and higher intentional-injury-weighted number of immigrants (*ViolentImmig*) induces higher homicide rates. These estimated coefficients appear to be important at conventional levels of significance across columns (2)-(7). Other covariates’ effects on homicide rate conform intuition and some are statistically significant, e.g., total rural population, population density, and industrial sector employment. Thus, the results in Table 2 roughly replicate the positive inequality-crime association found in many existing studies, albeit the relationship is not statistically significant in general.

Based on the results in Table 2, we add poverty level and average income in the model and the results are shown in Table 3. The results in columns (1)-(3) suggest a clear adverse effect of a greater degree of poverty (*Poverty*) and higher income level (*LnAvgInc*) of a prefecture appears to raise homicides there. However, columns (4)-(6) imply that the same poverty and income-level factors are insignificant but positive after controlling for the dispersion of income and other demographic covariates. Inequality in the tails (*DLnInc9505* and *DLnInc9010*) still show the significant and even larger values compared to Table 2. Likewise, only the coefficient of *DLnInc9010* is significantly positive which means an increase in income dispersion at 90th and 10th percentile income inequality would increase the homicide rate significantly. Therefore, our main results in Table 3 attest that all the poverty level, income-level, and income-dispersion factors show an adverse effect in homicide rate in prefecture-level. Moreover, the poverty-level and income-level appear to have more important and larger impacts than income inequality.

As for the control variables in Table 3, we find that the expenditures on policing (*LnPolicing*) reduces homicides significantly across columns (1)-(3) but changes to be positive and insignificant when adding inequality measures and other demographic covariates. This may arise from the other factors such as population, unemployment and income inequality play larger roles in homicide than public security expenditure. Similarly, the net inflow of poor migrants (*LnImmigPoor*) becomes positive on the homicide rate after adding other poverty and income inequality variables. The small and statistically insignificant effect of *LnImmigPoor* does not support the claim that the local homicide rate is largely determined by the poverty of net inflow migrants. However, this is not to say that violent crime is not related to internal migration, indeed, our variable, *ViolentImmig* which captures the spatially weighted influence of violence propensity due to the net inflow of migrants, positively contributed to the local homicide rate. Thus these results indicate that it is the violence per se that imported through migrants, rather than imported poverty, that escalates violent crime in a prefecture.

In order to conclusively decide on an appropriate factor closely related to homicides, we investigate further by comparing the difference between rural and urban areas of prefectures. Table 4 gives us more complicated results based on Table 3 by dividing key explanatory variables into urban and rural areas.

Relative to the insights from Table 3, we find that the same poverty, income-level, and income-dispersion factors matter when considered in urban and rural areas, respectively. A higher degree of poverty in the rural areas (*PovertyRur*) of a prefecture on homicides there, and also a higher income level in the urban areas (*LnAvgUrbInc*) of a prefecture appears to raise homicides there. However, columns(4)-(6) imply that a greater dispersion of incomes does not—neither in the urban nor the rural part of a prefecture—increase homicide rates in the prefecture. Hence, these results attest to the fact that poverty in the rural parts to be a push factor and higher income levels in the urban parts to be a pull factor of homicide rates. However, they do not attest to an important adverse effect of income inequality on prefecture-level homicides.

Furthermore, the effects of control variables reported in Table 4 are consistent with those in Table 3, implying the robustness of our estimation. The latter findings suggest that a negative impact of college education (*CollRate*) on homicide rates, and a positive impact of a higher degree of unemployment rates (*UnempRate*) on homicides. Both urban and rural population decrease homicide rates as shown by the negative and statistically significant coefficients of *LnPopUrb* and *LnPopRur*, which is in contrast to the empirical patterns observed in Latin American countries [71, 72].

We also do a robustness test by replacing the income inequality measures into the Gini coefficient (*Gini*) in Table 5. All columns in Table 5 show that a higher Gini coefficient (foremost in urban but even in rural areas) of a prefecture, regardless of overall, rural, or urban, reduces homicide rates, negating the inequality-crime nexus. At the same time, a higher poverty level, particularly in rural areas (both in terms of statistical significance and magnitude), drive up homicide rate. For the pull factor, a higher average income-level (particularly in urban areas) increases homicide rates. These findings are manifest the robustness of our benchmark results.

In the remainder of the discussion, we focus on the effects of log urban average income (*LnAvgUrbInc*) and rural poverty rates (*PovertyRur*) on homicide rates, using nonlinear (fractional-logit) predictions of the homicide rate in column (7) of Table 4.

**Fig 1:**
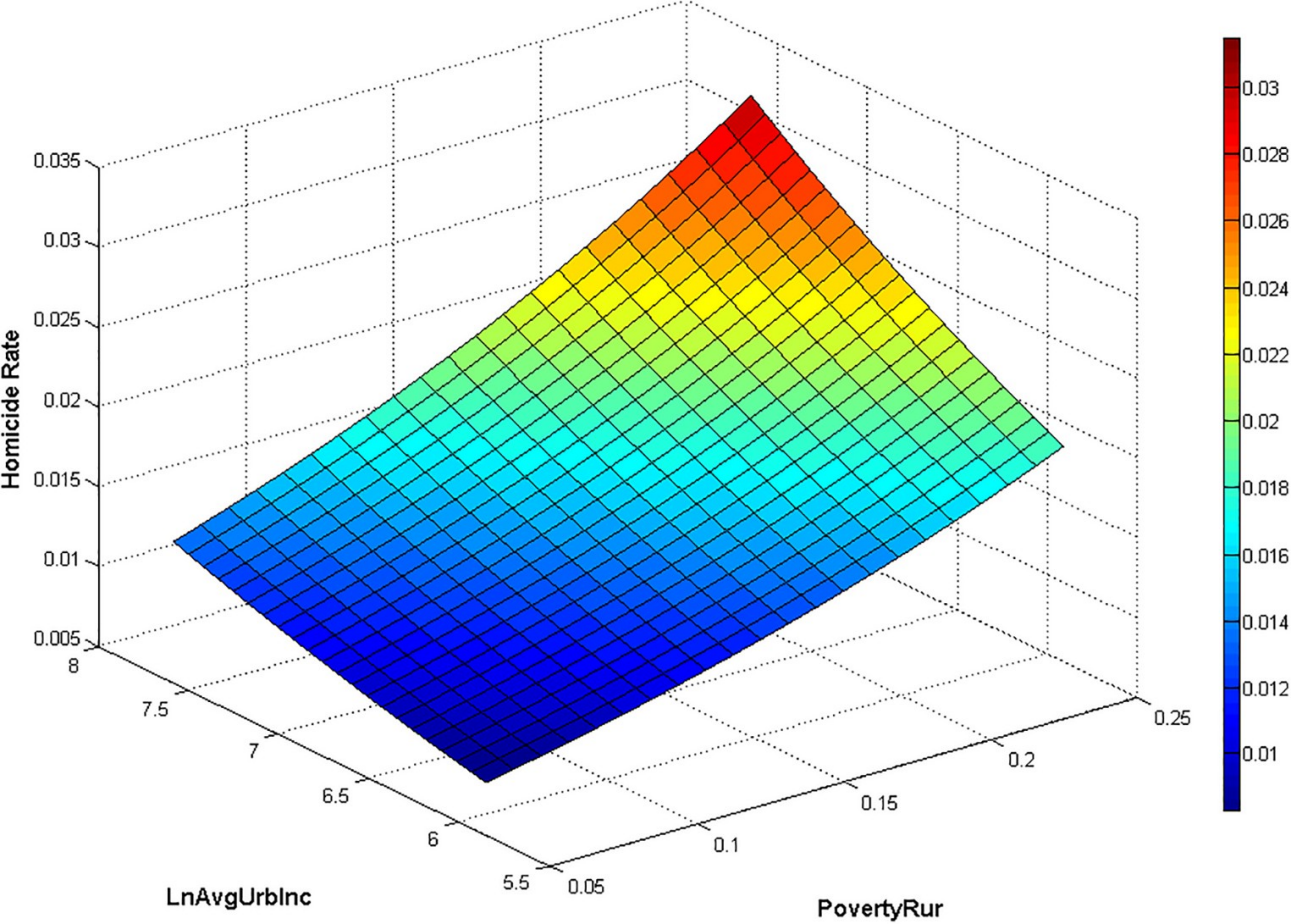
Predictions of homicide rate for the span of values of rural poverty and urban average income in the sample.

In Fig 1, we plot predicted homicide rates when evaluating all variables except *PovertyRur* and *LnAvgUrbInc* at their sample average. For generating the figure, we use a 21 × 21 grid of equally-spaced cells in the maximum-to-minimum range of *PovertyRur* and *LnAvgUrbInc* in the data. Consistent with the fractional-logit form, this obtains a three-dimensional nonlinear (S-shaped) function. The steepest ascent of this function would be in the neighborhood, where *Homicide* ≈0.5 with respect to *LnAvgUrbInc*, and 0.66 with respect to *PovertyRur*. However, the data are situated around a much lower mean. Accordingly, the three-dimensional nonlinear function is ascending in *PovertyRur-LnAvgUrbInc*-space throughout the sample. Hence, the steepest ascent in either dimension is at the maximum value of both *PovertyRur* and *LnAvgUrbInc* at the point where *Homicide* is highest in Fig 1.

**Fig 2:**
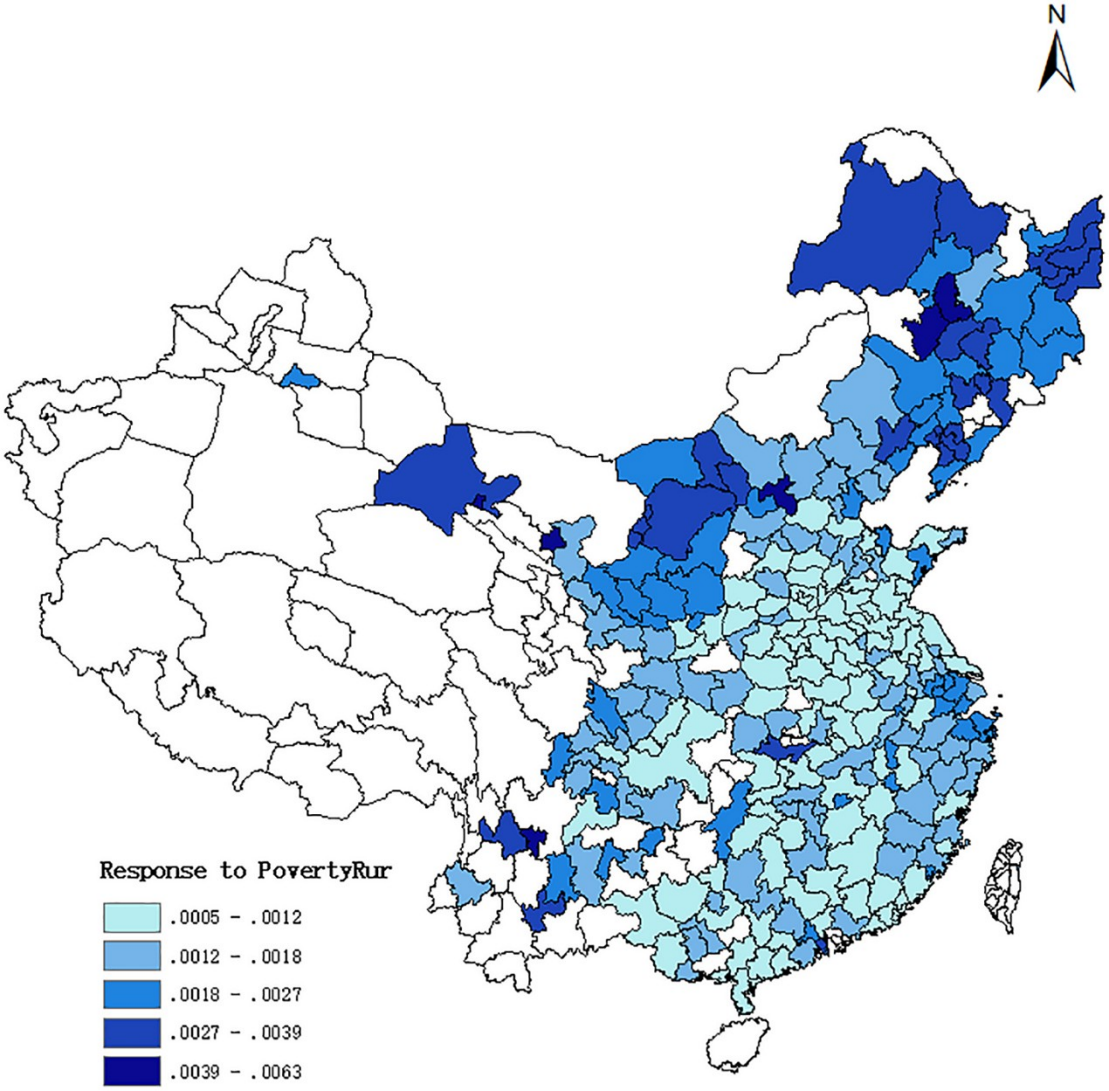
Response of homicide rate to a one-std.dev. Increase in Rural Poverty.

**Fig 3:**
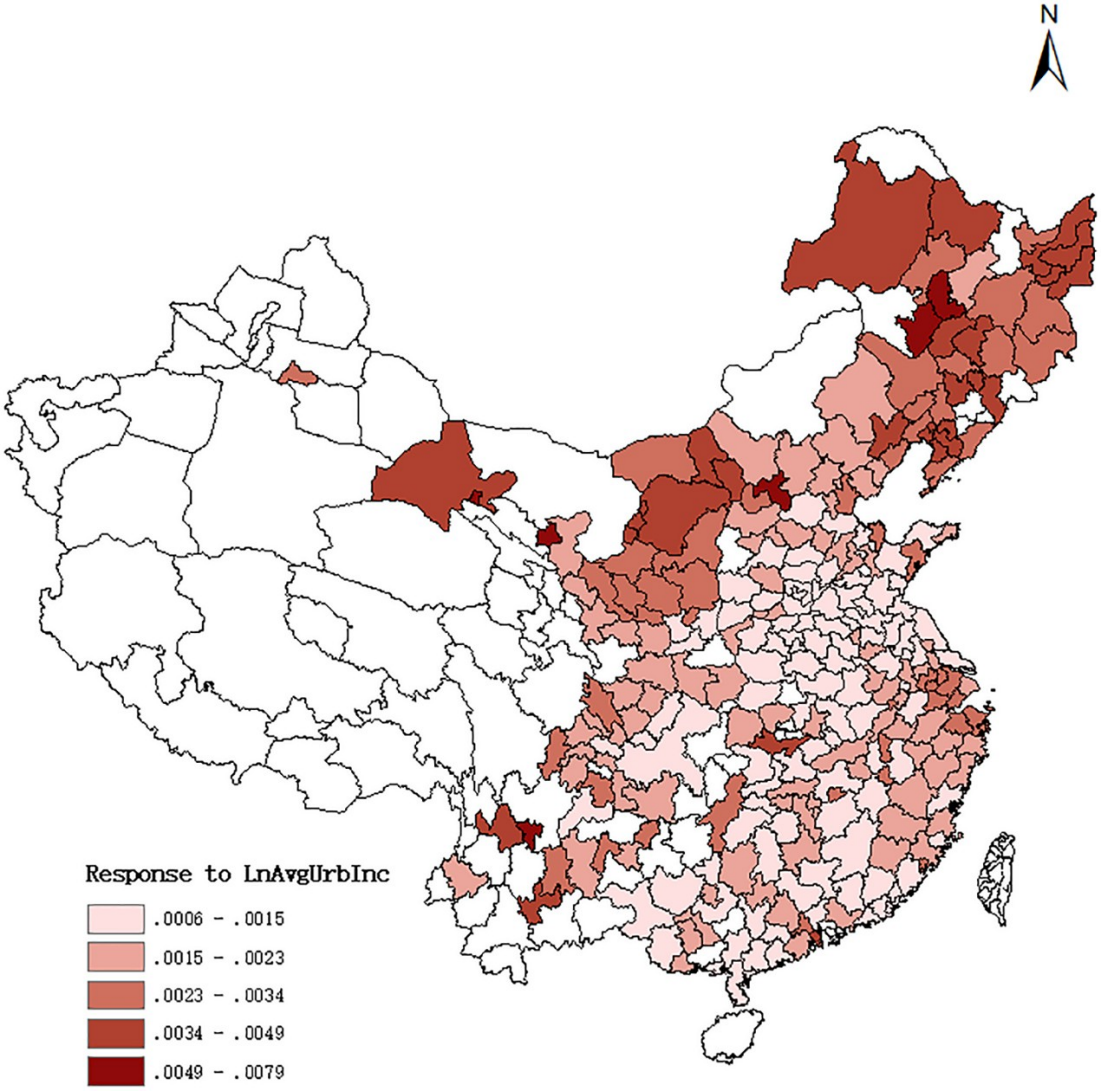
Response of homicide rate to a one-std.dev. Increase in Urban Average Income.

In Figs 2 and 3, we plot the marginal effects of these two variables for each prefecture in the data by way of a map. In each figure, we increase the respective variable by one standard deviation from the value a prefecture had in the outset. In this exercise, we do not use the means of all the other control variables, but we account for their prefecture-specific values. Fig 2 is devoted to the response of homicide rates to an increase in rural poverty, and Fig 3 to an increase in average urban income. The two figures suggest that there is a large degree of heterogeneity in the responses, and the effects tend to be largest in the northern prefectures and lower in the central and southern parts of China. In general, the effect of *PovertyRur* is much larger (and also its level of statistical significance is higher, which is not obvious from the maps) than that of *LnAvgUrbInc*.

## Conclusions

There is a burgeoning literature on the relationship between income inequality and violent crimes. The majority of this line of literature attributes the widened inequality as a driven force of higher violent crimes. However, the relationship may be spurious once some poverty measure is added, as argued by a handful of studies. The current paper provides support to the latter argument using court convictions on homicide throughout China, mini-census data on incomes, and migration survey data, all at the prefecture level.

We find that it is the poverty level in rural areas and the average income level in urban areas, rather than income inequality, that contribute to the local rate of incidence of violent crimes in China. This finding is robust against different measures of inequality and different empirical formulations. Apart from poverty in rural areas, the ‘transferred’ violence by internal immigrants pushes the crime rate.

The current paper therefore contributes to the ongoing literature by providing prefecture-level empirical evidence from China. Our study calls for a reexamination of the robustness of the results in earlier work by adding poverty measures in the regressions. The inequality-homicide association appears to likely disappear and the poverty-homicide association to hold, at least in China. Our purpose is not to argue that inequality is unimportant, but when it comes to violent crime, “absolute deprivation” appears to matter more than “relative deprivation”.
